# 

**Published:** 1883-07

**Authors:** 


### EDITORIAL.

### Meeting of the Board of Examiners.

At the meeting of the State Boards of Dental Examiners, held in Lexington, Ky., in February last, it was decided to hold the next meeting of that body in the Cataract House, at Niagara Falls, on Monday, August 6th, 1883, at 2 o'clock P. M.

It is desirable and important that Boards of *all the States* having laws regulating the practice of dentistry should be as fully represented as possible. Various matters of importance will be before the meeting, prominent of these will be the consideration of the propriety of adopting uniformity of procedure, so nearly as practicable.

Let the Boards of every State be as fully represented as possible.

### Pennsylvania State Dental Society.

The Pennsylvania State Dental Society will convene at Cresson Springs, on the main line of the P. R. R., July 31st, 1883, at 10 a. m., and continue in session four days. For all information address, W. H. FUNDENBERG, COR. SEC.

330 Penn. Ave., Pittsburgh, Pa.

### American Dental Association.

By official notice on another page it will be seen that the twenty-third annual meeting of the American Dental Association will be held at Niagara Falls, Tuesday, Aug. 7th., beginning at 10 o'clock A. M.

This is a meeting in which the entire profession of the country is more or less interested.

Its proceedings are worthy of attention in many respects. They are a pretty clear index of the development and growth of the profession. They exercise somewhat of a molding influence upon the various great interests of the profession. None can afford to ignore or disregard the clearly expressed declarations of this body.

The work of the association is varied. It consists of a series of sections, to each of which is assigned particular duties. It is a question whether the work of these sections has been carried out in strict accordance with the original plan. The work seems in many instances to have been performed by individuals and these rather few in number.

The plan originally contemplated was that the assistance of each member of the section should be rendered in the performance of the work, it might be but little in many cases, but the small efforts, if definitely made, would in the aggregate produce great results.

We will hope to have the reports this year, more definitely, that of the sections than heretofore.

Another work in which the association could profitably employ part of the time of its sessions, is the consideration of some of the more permanent interests of the profession, for instance, *education, society work, legislation, &c.* The annual consideration of these subjects would be productive of much good, if conducted in a proper manner and spirit. There ought to be a full report upon each of the subjects above named.

Let the approaching meeting be better and more efficient than any that have preceded, and if every member will have and manifest interest, and do what he can, such a result will surely be attained.

---

THE American Dental Association will meet at Niagara Falls the first Tuesday in August, at 10 A. M.

The Committee on Credentials, and the Treasurer, will be at the place of meeting at 8, A. M., Tuesday, at which time it is hoped the members and delegates will present their credentials and pay their dues, as far as possible, before the hour for regular meeting.

The afternoon of Tuesday will be set apart for the meeting of the different sections, to enable them to complete their reports to be presented to the General Association. J. N. CROUSE,

Chairman Executive Committee.

NOTICE TO STATE BOARDS OF DENTAL EXAMINERS.—There will be held, at the Cataract House, Niagara Falls, on Monday, August 6th, 1883, at 2 o'clock p. m., a meeting of all the State Boards of Dental Examiners, for the purpose of perfecting the organization of a National Association of Examining Boards. It is hoped that every Board will be fully represented.

GEO. H. CUSHING,
Sec'y of Conference held at Lexington, Ky.

### Ho! for Niagara.

The American Dental Association meets at Niagara Falls on Tuesday, Aug. 7th, 1883. The method of getting there is always a matter of some interest. All those who go from or through Cincinnati, have the choice of two routes at about the same rates, viz: the C. C. C. & I., via Cleveland & Lake Shore, or the N. Y. P. & O., via Chautauqua and Buffalo, at $14.75 the round trip either way. The trains by the former leave the Grand Central Station, Cincinnati, at 8 A. M., daily through car to Buffalo, arriving at Niagara Falls at 10 P. M. same day; and at 9 P. M., daily, arriving at Niagara Falls at 3 P. M. next day.

Trains on the N. Y. P. & O., leave the C. H. & D. R. R. Station at 1:45 P. M., and arrive at Niagara at 9 A. M. next day; and at 9:20 P. M., arriving at Niagara next evening at 6 o'clock.

Each of these routes has its advantages and special inducements, and persons going can take either without wishing they had taken the other. The tickets are good for return till October 28th.

Those wishing to go down the St. Lawrence to Montreal, Quebec, and White Mountains, can make arrangements for such an excursion either in Cincinnati or at Niagara Falls at greatly reduced rates. For any further information address Editor of the REGISTER.

AMERICAN DENTAL ASSOCIATION.—The twenty-third Annual Meeting of the American Dental Association will be held at Niagara Falls, commencing Tuesday, Aug. 7th., 1883.

GEO. H. CUSHING, REC. SEC'Y.

### CONTENTS.

### THE DENTAL REGISTER,

A MONTHLY MAGAZINE DEVOTED TO THE INTERESTS OF THE

**DENTAL PROFESSION.**

*Terms Reduced to* $2.00 *Per Year. Single Copies,* 25 *Cents.*

**EDITED BY PROF. J. TAFT, D.D.S.**

—AND—

***Published by SPENCER & CROCKER, Ohio Dental Depot.***

The volume commences in January and closes in December.

The REGISTER being issued monthly is always fresh, therefore Indispensable to the practitioner who desires to keep posted up with the new inventions and improvements which are daily developed. Neither expense nor effort will be spared to give value and variety to its contents. Articles will be illustrated with well executed engravings whenever they will add to the interest or assist to explanation.

Its extensive circulation and frequency of issue make it one of the BEST DENTAL ADVERTISING MEDIUMS in the United States.

All subscriptions must begin with January or July.

Local or special notices, five lines or less, will be inserted for $3 each insertion; over five lines, 50 cts. per line.

All advertising bills payable in advance, or at furthest, after the issue of the first number containing the advertisement. All transient advertisements to be paid for in advance.

☞CONTRIBUTIONS SOLICITED.

Exclusively Editorial Communications should be addressed to the Editor.

The latest number of the REGISTER may be ordered with or without other goods at any time, and all letters should be addressed to

**SPENCER & CROCKER, Ohio Dental Depot, Cincinnati, O.**

